# Dr. Amos P. Dodge

**Published:** 1916-07

**Authors:** 


**Dr. Amos P. Dodge,** University of Maryland, 1874, died at his home in Oneida, N. Y., May 3, aged 63. He settled in Oneida Castle in 1876 and has resided in that vicinity ever since.

